# A sensitive soil biological indicator to changes in land-use in regions with Mediterranean climate

**DOI:** 10.1038/s41598-022-26240-9

**Published:** 2022-12-23

**Authors:** Yosef Steinberger, Alfred Stein, Michael Dorman, Tal Svoray, Tirza Doniger, Oshri Rinot, Eshel Gil

**Affiliations:** 1 The Mina and Everard Goodman Faculty of Life Sciences, Bar-Ilan University, 5290002 Ramat Gan, Israel; 2 Faculty of Geo-Information Science and Earth Observation, University of Twente, PO Box 217, 7500 AE Enschede, The Netherlands; 3 Department of Geography and Environmental Development, Ben-Gurion University of the Negev, Beersheba, Israel; 4 Institute of Soil, Water and Environmental Sciences, Agricultural Research Organization, The Volcani Center, Rishon LeZion, Israel; 5 Soil Erosion Research Station, Ministry of Agriculture and Rural Development, HaMaccabim Road, P.O. Box 30, 50200 Rishon LeZion, Beit Dagan, Israel

**Keywords:** Ecology, Environmental sciences

## Abstract

The demand for reliable indicators to quantify soil health has increased recently. We propose and test the use of soil microbial functional diversity as an indicator of multifunctional performance in agriculturally important areas. Agricultural fields in the Mediterranean and semiarid regions of Israel were selected as test sites and measured in Spring and Autumn seasons. Measurements included microbial parameters, basic soil abiotic properties and biological responses to agricultural management relative to measures of a natural ecosystem. Using a canonical correlation analysis we found that soil moisture was the most important basic soil property with different responses in Spring and Autumn. In Spring, it had a strongly negative relation with microbial biomass (MB), community level physiological profiling (CLPP) and the Shannon-Weaver index H', while in Autumn it had a strong relation with CLPP. We further show a significant interaction between CLPP and climate for land-use type "orchards". CLPP measured in the autumn season was thus identified as a useful and rapid biological soil health indicator, recommended for application in semiarid and Mediterranean agricultural regions. Apart from obtaining a better understanding of CLPP as the soil indicator, the study concludes that CLPP is well suited to differentiate between soils in different climates, seasons and land use types. The study shows a promising direction for further research on characterizing soil health under a larger variety of conditions.

Long-term agricultural activity may lead to substantial changes in several chemical, physical and biological soil properties and to the overall soil health status^1,2^. To be able to estimate those changes, there is a burning need for reliable indicators that are sensitive to changes in land-use and land management, and, at the same time, are cost-effective^3^. This challenge is even larger in the case of biological soil health indicators^4–6^ that represent the soil biota activity, composition, density, diversity and trophic interactions.

Due to this complexity and the intricacy that non-linear interactions of biological processes cause to the development of soil indicators, most studies on soil health are focused on chemical and physical processes^7–10^, with less emphasis on the biological compartments. As the knowledge on soil biota community and the methods to evaluate their contribution is increasing, more emphasis should be placed on soil biological role and functions. The increasing understanding of the importance and the effect of soil biological properties on soil health will increase our capability to draw a comprehensive soil health index.

The literature provides several papers on soil health and biological indicators. One of the first papers^11^ used biological indicators of soils to analyse their functional stability and substrate utilisation following environmental impacts. Along a similar line, soil health in an Italian polluted site was characterized using microorganisms as bioindicators^12^. In a slightly different context, biological indicators were used to address the soil health of banana plantations^13^. Larger areas were addressed in subsequent papers^14,15^, where landscape scale surveys were reported on indicators of soil health in grazing systems, and a regional analysis was carried out, reporting Statistics and Scoring Functions, being contained in a comprehensive Soil Health Database. Somewhat as a surprise, in a study in the US it was found that soil health indicators do not differentiate among agronomic management systems^16^. Finally, the potential for biological soil health assessment was discussed recently by^17^, who chose a ratio of principal components to measure soil health.

In this study we will be using soil microbial biomass; soil respiration; community level of physiological profiling (CLPP) based upon substrate-induced respiration (SIR). In the past is was suggested to represent the soil microflora activity, e.g., by bacteria and fungi^8,18,19^. Such an attempt, however, should be taken with care, as soil microbial diversity fulfils its functioning in supplying nutrients and stable ecosystem services, while it is shaped by edaphic characteristics on which human society is highly dependent. Of particular interest is the non-balanced input–output of organic matter in different land-uses: they substantially affect soils in their long-term organic carbon content, nitrogen content and other components as a contribution to the soil^20,21^. The soil microbial community is either the mediator, or the eye of the needle. For example Dwivedi and Soni have found that soil organic matter is the energy source of that community^22^.

Within this context, any current attempt to explore the use of biological soil health indicators relies on two questions: 1) which basic biological soil properties of the aforementioned plethora are most sensitive to changes in land-use?; and 2) assuming seasonality plays a major role in soil biota community composition and activity, then what is the best season for soil sampling?

The objective of our study is to determine a simple and cost-effective biological indicator sensitive to changes in land-use. We chose the synergy of a biological component with abiotic variables, that were previously reported as most sensitive to detect soil variation in properties due to agricultural management^23^. We tested the indicator in two seasons.

## Methods

### Site description

**Figure 1:**
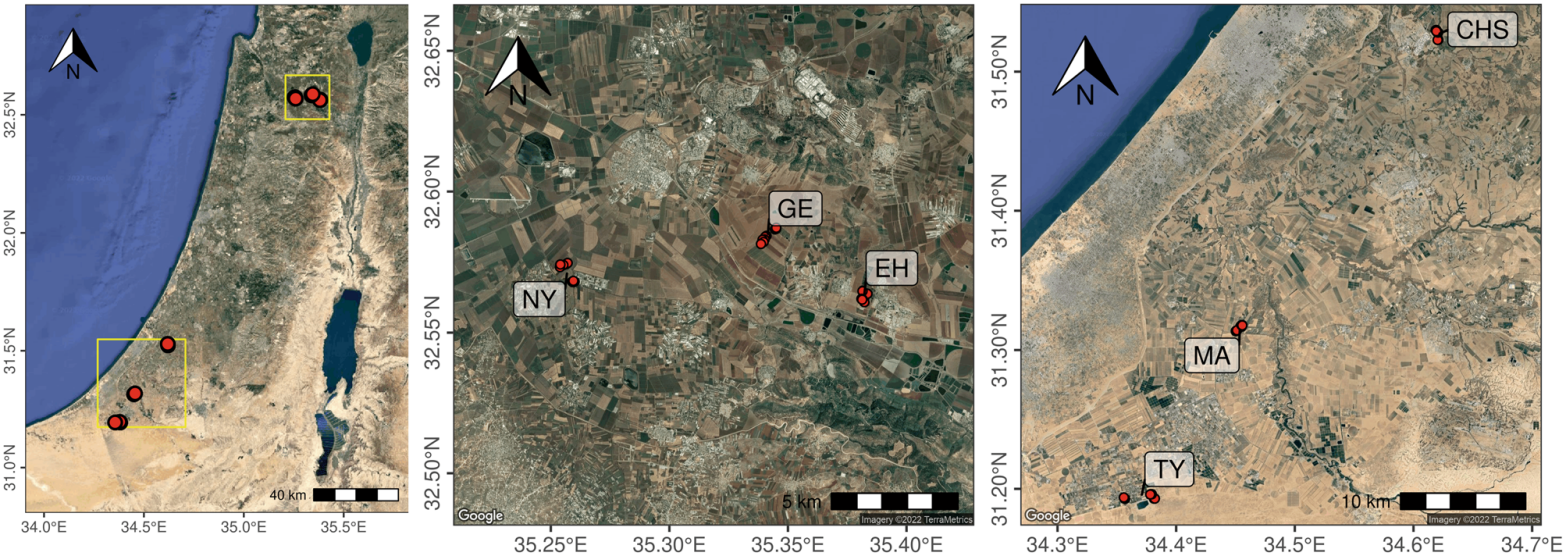
Maps of the study sites in Israel. The Yizrael valley has three sites Ein Harod (EH) Geva (GE) and Nir Yafe (NY); three sites Magen (MA), Chavat HaShikmim (CHS) and Talmey Yosef (TY) were located in the north-west part of the Negev.

Soils were sampled in two climatic regions in Israel, during two seasons: (i) Spring, or late winter; and (ii) Autumn, or late summer. The two regions are characterized by wide and intensive agricultural systems with three common management practices, demonstrating different climatic conditions and soil textures. Three sampling sites Ein Harod, Geva and Nir Yafe were located in region 1, the Yizrael valley, having a Mediterranean climate and clayey soils. Three other sites Magen Chavat HaShikmim and Talmey Yosef were located in Region 2, at the north-west part of the Negev, having a semi-arid climate and silty to sandy soils (Fig. 1).

Sampling campaigns were carried out in the two seasons to represent contrasting soil moisture content. Spring sampling was conducted in February 2017 in the Yizrael valley and in February 2018 in the Negev desert, following a winter season that was characterized by annual precipitation of ~ 400 mm, leaving moist soil samples. Autumn sampling was conducted in both areas in September–October 2016, following a warm and dry summer, with temperatures 28 °C on average, and without rainfall. Three representative plots with different land-uses were selected in each region. Soil types were Typic Chromoxererts in Region 1, and were Vertic Calcixeralfs, Typic Haplargids and Typic Quarzipsamments in Region 2^24^. Land-use types were orchards (OR), field crops (FC) and a control plot (CO–non-cultivated plots). Orchards, representing perennial crops, were routinely irrigated to provide optimal water content for plant growth, while the field crops were irregularly irrigated during the growing season. The plots were cultivated under the same land-use type at each plot for the last 15 years. The selected plots were as close as possible to each other, i.e., within a range of 2 km. During summertime, field crop plots were left exposed fallowed, i.e., without vegetation.

Soil samples were collected at three depths along the 1 m soil profile (0–10 cm, 10–30 cm and 30–60 cm) using 3 samples taken in each of the *n* = 18 sampling pits. From each soil sample, approximately 1 kg of soil was immediately sieved by a 2 mm sieve, kept in a sealed bag, and placed in a cooling box (4 °C) for further analyses. It provided us with a dataset of 162 observations for each season.

### Soil analysis

A range of different soil measurements was taken. Below these are listed providing a detailed and technical description.

Three basic soil variables were obtained: soil moisture, soil salinity and soil pH. *Soil moisture* (SM) content was determined gravimetrically by drying soil samples for 24 h at 105 °C. The dry soil was used to estimate *organic matter* (OM) content using a muffle furnace at 390 °C for 8 h. *Soil salinity* (EC) was determined as the electrical conductivity (EC) of 1:2 (soil: distilled water) extracts—by an auto-ranging EC/temp meter (TH2400, EI-Hamma). *Soil pH* was determined with a pH electrode in the filtered supernatant after an overnight incubation period at room temperature of a mixture containing 20 g soil and 40 ml distilled water (1:2 soil:water ratio), followed by shaking for 10 min (160 rpm) and incubation overnight at room temperature. In addition, *Soil Carbon and Nitrogen were determined:* Dried soil samples were used to determine the total C and N content by using a C:N analyser (Flash EA 1112 series, Thermo). The OC was measured indirectly by subtracting the inorganic C as determined by a calcimeter from the total C. The ratios between organic C and nitrogen were then calculated.

A different set of variables was determined in order to characterize soil health and soil quality, namely CO_2__Ev (Evolution) was detected using the MicroRespTM plate method^25^. The standard procedure runs as follows. Equal volumes (25 μl) of glucose and distilled water were then added separately to another four deep wells to determine active soil microbial biomass (MB^26^ and CO2 evolution (CO_2__Ev), respectively. CO_2__Ev was measured by dye plates, being a colorimetric reaction that uses absorbent alkali with the ability to measure CO_2__Ev released from each well, with a spectrophotometer at 590 nm^26^. The plates were read twice: just before (t_0_) and one hour (t_1_) after being placed on the deep well plates containing the soil samples and carbon sources. Such a short interval was chosen to have highest data reliability^27^. During this period, the plates were incubated in the dark at 25 °C. The result for each well was determined based on the 16th well, which contained only water with the soil sample, and was measuring CO_2_ evolution with no additional carbon source.

MB was estimated by adding glucose to the samples and converting glucose-induced respiration rates. In both cases the CO_2_ evolution was determined by detection system—(colorimetric gel detector plates -cresol red) and an automated plate reader^25^. Colorimetric gel detector plates were filled with 1% Noble agar (150 μl well^-1^) containing a pH indicator dye, cresol red (12.5 μg g^-1^ wt wt^-1^), 150 mM potassium chloride, and 2.5 mM sodium bicarbonate.

Substrate utilization profiles and community-level physiological profile (CLPP) in the soil were also measured by this system. The modified MicroRespTM method was used to construct sole-carbon-source utilization profiles of soil microbial communities. Fifteen different carbon sources belonging to one of the four carbon groups: 1) aromatic carboxylic acids; 2) carboxylic acids; 3)carbohydrates and 4) amino acids were added to soil samples in deep well plates.

The different carbon sources added in soil in the MicroResp detection system were : Aromatic carboxyllic acids—3,4-Dihydroxybenszoic acie (protocatechuic acid); Carboxylic acid—L-Alanin, Arginine, L-Cysteine HCl, g-Amino butyric acid, L-Lysine, N-Aceyl-glucosamine; Carbohydrates—L-Arabinose, D-Fructose, D-Galactose, D-Glucose, Trehalose; Amino-acids—Citric acid, L—Malic acid, Oxalic acid. Amounts of 20 g soil from each soil sample were then incubated for 48 h in the dark at 25 °C and at 40% of their water-holding capacity. Twenty-five μl of the eight carbon sources and distilled water (blank) were respectively dispensed into deep well plates, and equal volumes of distilled water were added to other deep wells to determine soil basal respiration, while the glucose was used to determine microbial biomass. The incubated soil samples were added to the substrate plates^25^, and the plates were left open for a period of 45 min to allow for the release of any carbonates present in the soils^27^. The respired CO_2_ was absorbed by the gel detection plates and measured using a spectrophotometer at 590 nm. The plates were read twice: just before and 1 h after being placed on the deep well plates containing the soil samples and carbon sources. During this period, the plates were incubated in the dark at 25 °C. Respiration rates were calculated from adsorption data, minus the well containing only water with the soil sample (blank). The results for each well were calculated based on the initial colorimetric value.

Microbial functional diversity was estimated using the well-known Shannon-Weaver index (H'): H’ =  − Σp_i_ (ln p_i_), where p_i_ is the ratio of the activity of a particular substrate to the activities of all substrates and summation is carried out over the activities of all substrates, indexed by i^28,29^. All soil data are available upon request.

### Statistical analysis

Statistical analysis was carried out to test the hypothesis that relations exist between soil biotic components and composition of the substrates. The statistical analysis consisted of a canonical correlation analysis followed by a regression analysis. A canonical correlation analysis provides a multivariate analysis allowing a global interpretation between sets of variables, in contrast to an individual correlation analysis that relates individual variables. A canonical correlation analysis determines linear combinations of the variables of the two sets, which have maximum correlation with each other^30–32^. The two sets of variables are denoted here as Set 1 and Set 2. Set 1 contains the observations on soil abiotic components, potentially serving as explanatory variables SM, OM, EC, pH, TN, OC and CN, whereas Set 2 contains the observations on the substrates potentially serving as response variables: CO2_Ev, H’, MB and CLPP (Fig. 1). All statistical analyses were applied separately for observations made in Spring and in Autumn. Missing data are treated such that any observation where a missing value on one of the variables occurs was deleted. Relations between the two groups are of a particular interest, while these relations were further evaluated using a linear regression analysis. Canonical correlation analyses were carried out using SPSS^33^; regression analyses were programmed in R^34^. Model selection was carried out using R package MuMIn^35^, maps and figures were produced using R package ggplot2^36^.

## Results

### Abiotic and biotic components

**Figure 2:**
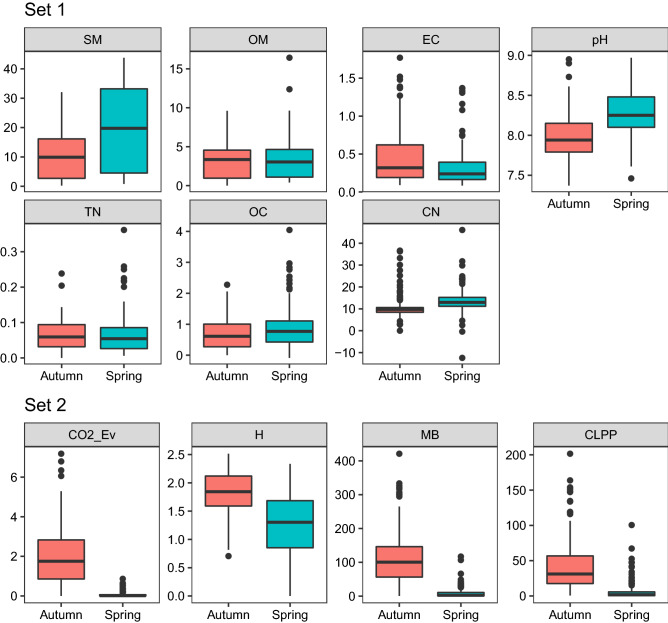
Distribution of soil sample properties for samples collected in autumn and spring. The properties are marked according to their role in the subsequent analyses as Set 1 variables and Set 2 variables. The solid line in each box indicates the data median, the boxes indicate the 1st and 3rd quartiles, whiskers extend to at most 1.5 times the size of a box, while observations above the upper whiskers and below the lower whisker are outliers.

A box plot descriptive analysis shows large differences in the Spring data for the Set2 variables (Fig. 2). It shows that CO2_Ev, MB and CLPP have little variation, with similar 1^st^, 2^nd^ and 3^rd^ quartiles. In Autumn, a higher variation is observed, indicating that in the dry season (Autumn), the characteristics of the substrates are much more distinguished than in the wet season (Spring).

### Pairwise Pearson correlations

**Figure 3:**
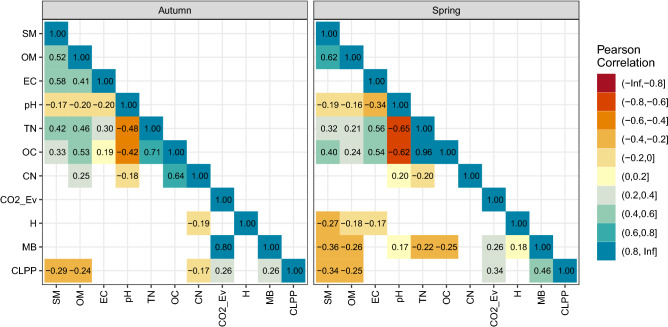
Pairwise correlation between soil characteristics, in each of two seasons. Text labels specify the Pearson correlation coefficient. Insignificant (*p* > 0.05) correlation coefficients are not shown.

Figure 3 shows the pairwise correlations between the variables in Set1 and Set2. In Spring, pairwise correlations reveal a strong link between OC and TN (*r* = 0.96). Such a high correlation within one of the Sets means that OC and TN provide the same information about the samples, which may affect the analysis, and hence we modified Set1 by excluding OC.

### Canonical correlations

Table 1 shows the canonical correlations between Set 1 and Set 2 in Spring. The first canonical correlation value (0.491) was higher than the second one (0.235) and was the only significant one (*p* =  < 0.001).

**Table 1:** Canonical correlations between the two sets of variables in Spring.

	Correlation	Eigenvalue	Wilks statistic	F	Num D.F	Denom D.F	Sig
*Canonical correlations*							
CV_1_	0.491	0.318	0.681	2.457	24	507.055	< 0.001
CV_2_	0.235	0.058	0.898	1.066	15	403.443	0.386
CV_3_	0.202	0.042	0.951	0.942	8	294.000	0.482

The first canonical variable (CV_1_) shows a significant correlation (*p* = 0.001), while the other canonical variables showed non-significant effects.

H_0_ for Wilks test is that the correlations in the current and following rows are zero.

We next turn to the interpretation of the canonical variables (CVs) in Set 1 (Table 2). The first canonical variable (CV_1_), the only significant one, scores high with respect to SM. SM hence serves as the variable in Set1 that provides the major distinction with Set2. We also inspected the non-significant second and third canonical correlation. The second canonical variable (CV_2_) showed a strong negative score for pH and to a somewhat lesser degree with TN, while the third canonical variable (CV_3_) scored high for TN, with a positive sign. We may therefore interpret CV_1_ as SM, CV_2_ as the joint contribution of pH and TN (with a negative sign), while CV_3_ is similar to TN. Note that TN was very closely related to OC, which may be important when it comes to interpretation.

**Table 2:** Standardized correlations in Spring between the four canonical variables in the different columns and the variables in the two sets.

Set1				Set2			
Variable	CV_1_	CV_2_	CV_3_	Variable	CV_1_	CV_2_	CV_3_
SM	0.812	0.194	− 0.216	CO2Ev	0.085	0.535	− 0.911
OM	0.176	− 0.009	− 0.162	MB	− 0.442	− 0.731	− 0.433
EC	0.315	− 0.207	− 0.595	CLPP	− 0.485	0.457	0.457
pH	0.168	− 1.118	0.479	H'	− 0.547	0.701	0.351
TN	0.025	− 0.509	1.384				
CNrat	− 0.021	− 0.252	− 0.423				

Turning to Set2 we see that CV_1_ had a strong joint correlation with MB, CLPP and H, all in the negative direction. This signified a strong relation between these three variables, while CO_2__Ev stood out and came only back in the, non-significant, CV_2_. Clearly, the complex indicated by MB, CLPP and H was the most important one in relation with the Set1 variables.

Focusing on the variables contained in CV_1_, we conclude that in spring there was a strong relation between SM and the relational complex between MB, CLPP and H. An increase in SM corresponded with a decrease in these three variables in Set2. The second (non-significant) CV_2_ pointed to a negative relation of CO_2__Ev and pH on the one hand and mainly MB on the other.

We next analysed the data collected in Autumn. There was no obvious reason to remove any variable as was done in the Spring sample. Table 3 shows the canonical correlations between Set1 and Set2. As in the Spring data, we note that the first correlation value (0.423) was the only significant one (Sig. = 0.014).

**Table 3:** Canonical correlations between the two sets of variables in Autumn.

	Correlation	Eigenvalue	Wilks statistic	F	Num D.F	Denom D.F	Sig
*Canonical correlations*							
CV_1_	0.423	0.218	0.730	1.772	24	451.273	0.014
CV_2_	0.279	0.084	0.890	1.038	15	359.274	0.415
CV_3_	0.165	0.028	0.964	0.599	8	262.000	0.779

H_0_ for Wilks test is that the correlations in the current and following rows are zero.

The first canonical variable (CV_1_) shows a significant correlation (*p* = 0.021), while the other variables showed non-significant effects.

The first canonical variable (CV_1_) scored highly negative for SM. We therefore interpret it as the (negative) amount of SM. All other variables have a much weaker correlation with CV_1_. Similar as for the Spring data, we also briefly inspected the second canonical variable. The non-significant CV_2_ is more difficult to interpret, mainly showing a distinction between OM and TN on the one hand and EC and CNrat on the other.

For Set2 we notice a positive score of CV_1_ with CLPP (Table 4). In Autumn, therefore there was a significant negative relation mainly between SM and CLPP. This being strongest, there are also relations with other variables related to these two. For instance, TN had a negative relation with SM, and hence showed a positive relation with CLPP.

**Table 4:** Standardized correlations in Autumn between the four canonical variables in the different columns and the variables in the two sets.

Set1				Set2			
Variable	CV_1_	CV_2_	CV_3_	Variable	CV_1_	CV_2_	CV_3_
SM	− 1.054	− 0.045	0.461	CO2Ev	− 0.062	0.006	0.061
OM	− 0.241	− 0.567	− 0.980	MB	− 0.381	− 0.384	0.906
EC	0.417	0.512	0.479	CLPP	0.986	0.258	0.184
pH	0.139	0.164	0.174	H'	0.277	− 0.896	− 0.343
TN	0.363	− 0.426	0.647				
CNrat	− 0.271	0.721	0.000				

The canonical correlation analysis showed that, for both Spring and Autumn, SM was the most important variable in Set1, but its relations were different in the two seasons. In Spring, it had a strongly negative relation with the MB, CLPP and H complex, while in Autumn it had a strong relation with CLPP. The major difference observed was that in Spring MB and H were jointly related with CLPP, while this relation was absent in Autumn. It indicated a rather important difference in soil processes between the two seasons.

### Regression

A model selection procedure based on AICc was carried out to move from identifying relations towards causality^37^. In each season, we evaluated the effect of SM on each of the Set2 variables (i.e., CO2_Ev, H’, MB and CLPP), resulting in 2 seasons × 4 variables = 8 models being evaluated overall. In each model, the independent variables were SM and SM squared (SM^2^), to evaluate potential non-linear effects of soil moisture on microbial activity. Using AICc, we selected the most parsimonious model from three candidate models:

$$ \begin{gathered} {\text{Y}}\, = \,{\text{Intercept}}. \hfill \\ {\text{Y}}\, = \,{\text{Intercept}}\, + \,{\text{SM}}. \hfill \\ {\text{Y}}\, = \,{\text{Intercept}}\, + \,{\text{SM}}\, + \,{\text{SM}}^{{2}} . \hfill \\ \end{gathered} $$

where Y was one of the four Set2 variables, and (1) represents the null model where SM has no effect on microbial activity, (2) represents a linear relation, and (3) represents a non-linear (quadratic) relation.

**Figure 4:**
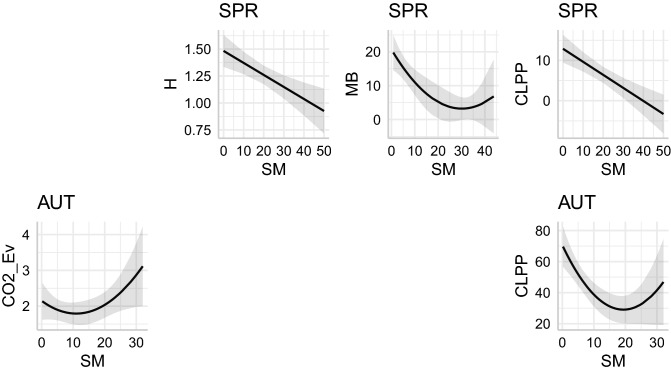
Effect of SM on CO2_Ev, H’, MB and CLPP in each of two seasons. Figures show predicted values based on the models listed in Table 5. In the two cases when the null model was selected (i.e., SM effect was not supported by the data), no figure is displayed.

According to the results (Table 5), SM non-linearly affects all four microbial properties in Spring, and CO2_Ev and CLPP in Autumn. In all cases, the response had a similar shape: minimal activity at intermediate SM and increased activity in high or low SM (Fig. 4). The analyses therefore indicated that SM explains the variation of a range of biological response variables in Spring, i.e., under wet conditions, while in Autumn in only affects CO2_Ev and CLPP.

**Table 5:** Effect of SM on CO2_Ev, H’, MB and CLPP in each of two seasons.

Season	Variable	Term	Estimate	std.error	Statistic	*p* value
SPR	CO2_Ev	Intercept	0.0744	0.0114	6.5488	< 0.001 ***
		SM				
		SM^2^				
	H	Intercept	1.4834	0.0757	19.5987	< 0.001 ***
		SM	− 0.0112	0.0032	− 3.5185	< 0.001 ***
		SM^2^				
	MB	Intercept	20.6855	2.9424	7.0301	< 0.001 ***
		SM	− 1.16	0.4589	− 2.528	0.0125 *
		SM^2^	0.0193	0.0118	1.63	0.1052
	CLPP	Intercept	12.8899	1.7514	7.3596	< 0.001 ***
		SM	− 0.3238	0.0734	− 4.4116	< 0.001 ***
		SM^2^				
AUT	CO2_Ev	Intercept	2.1499	0.2714	7.923	< 0.001 ***
		SM	− 0.0651	0.0464	− 1.4024	0.1631
		SM^2^	0.003	0.0016	1.8054	0.0732
	H	Intercept	1.822	0.0344	52.963	< 0.001 ***
		SM				
		SM^2^				
	MB	Intercept	113.3087	6.7294	16.8378	< 0.001 ***
		SM				
		SM^2^				
	CLPP	Intercept	70.5674	6.7821	10.4049	< 0.001 ***
		SM	− 4.284	1.1609	− 3.6903	< 0.001 ***
		SM^2^	0.1106	0.0411	2.6885	0.0081 **

In each of the eight cases, the terms included in the best model (i.e., lowest AICc) are given in three separate rows (Intercept, SM, SM^2^). Where a given term was not included in the best model, the row was left blank.

This analysis essentially confirmed what we identified earlier from the canonical correlation analysis, but, adding quantitative values and causality to the observed relations.

## Discussion

Our research objectives included identification of soil biological indicators sensitive to land-use and to identify the preferred season to sample the soil in the field; analysis of the relations between abiotic and biotic soil components; and deriving a simple method for evaluating soil condition. The conceptualization and the methodology followed by validation^10^ was based on natural soil productivity and agricultural management. Zhuo et al. however, proposed a cost-effective approach based on primary production assessments and taking into consideration the soil organic matter as the only soil property in their soil health index^10^. The natural system, however, is based upon short-term inputs that cannot be predicted in time and amount. In contrast, agricultural ecosystems are either based on continuous nutrient and water input or rely on pulsed nutrient and irrigation inputs at specific times and locations in the pedon to meet crop needs^38^.

When comparing Autumn with Spring, we found that the biggest stress period helps to separate between land-uses: different land-use types obtain different amounts of water in the two seasons. Moisture supply is optimal in spring, masking the differences in soil health status, while, in the much drier autumn period, much clearer differences are observed. By considering two extreme conditions, we show that the thermal and hydrological regime within the soil physics-soil chemistry interplay determines the components and the activity of soil biota with emphasis of microbial community composition, diversity and activity in our case. These conditions varied and were unpredictable in time and space, in particular for what we consider as the two common most important measurable variables: microbial biomass and CO_2_ evolution. Therefore, taking their cumulative quantitative data into consideration will help to come to a more sensitive determination of soil health statue.

Soil moisture levels are an important parameter determining biomass production that provides potential food for soil biota in general and for microflora in particular. Decrease in moisture availability inhibit microbial activity by lowering intracellular water potential by reducing hydration activity of enzymes and mobility. Moreover, decrease in moisture below a certain level will reduce diffusion of soluble substrates. Temperature plays an important role in cell denaturation stages and functional pathways. Were both moisture and temperature control carbon and nitrogen mineralization—therefore both factors—are important environmental parameters in determining seasonal changes. High moisture content corresponds with low CLPP low heterogeneity, i.e. high homogeneity in substrate utilization. Following the decrease in moisture content the CLPP is increasing where utilization heterogeneity is increasing. The difference between the two is towards enhancing a higher diversity. Such a change may emphasize the importance of autumn sampling.

The soil microbial community is an abundant and dominated community associated with organic matter that is present throughout the soil profile in natural and manmade management systems^39^. To receive valuable data on microbial community complex interactions with the environment are important. These complex interactions are being represented by the driest (autumn) and moist (spring) environmental conditions, from which the microbial community cannot escape^40,41^. However, microbial communities can inactivate during times of stress. To further address this issue, additional research is required. We also did not consider other forms of soil biota such as fungi or nematodes, nor did we separate the different kinds of microorganisms. Long term studies documenting microbial community activity in natural and agroecosystem following disturbance require a minimum data set to elucidate soil health status.

Soil biological activity, biomass, CO_2_ evolution and CLPP, are affected by various anthropogenic influences, namely human activity substrate availability and agricultural management^22,42,43^. As soil systems can be viewed as living systems, the two main measurable components are changes in microbial community biomass and respiration. These reflect changes in soil physico-chemical conditions^44^, restoration^45^ and primary production^46^. Hence, these can be used as soil health indicators. In this sense, other measurements are related to these factors.

A central element of the current study is a solid statistical analysis of collected data. Such an analysis is invaluable to analyse a large and complicated dataset as the present one. Traditional methods like canonical correlation and regression analysis were the chosen methods. This choice was based upon the nature of the data. For instance, we considered a classification to be less interesting, as the classes Season, Landuse and Climate were already present, and the main interest was in identifying the relations between the variables in those classes.

Soil moisture was found as one of the main triggers that affects microbial abundance and activity rate^19,47–51^ as obtained here. The contribution of soil moisture at the sampling time in understanding the soil health status supports our suggestion that the preferred sampling period is the autumn in comparison to spring season. Wardle and Parkinson in their classical study^52^ elucidated the strong correlation between soil moisture and soil microbial biomass and substrate utilization. Soil moisture, depending upon unpredictable input variable such as rainfall increase spatial variability and dissimilarity between the different agro-ecosystems according to the crop management, increase in spring relation between MB and CLPP. The autumn season in Mediterranean regions brings the abiogenic stimulants—in natural as well in the agro-management fields to similar status at the end of harvesting and before the field preparation for the new seedling—growing season. The autumn measurements of microbiota activity represent the effect of land-use per-se by its variability, rather than the spring season which is strongly affected by a wide range of anthropogenic-multivariate effects. The present study elucidates large differences in microbial biomass, CO_2_ evolution and substrate utilization among dry and moist seasons, and the importance of choosing the Autumn season for measurements, when moisture content is low and microbial community is limited by soil health properties we are interested in quantifying.

## Conclusions

From the results of this study, we conclude that soil moisture drives the different processes and hence serves as a proxy for biological soils' indicators. Both the canonical correlation analysis and the subsequent regression analysis indicate strong relations of Soil Moisture with Community Level Physiological Profile (CLPP) alone in Autumn, and with CLPP, Microbial Biomass (MB), and the Shannon-Weaver index (H') in Spring. Soil moisture significantly affects CLPP in both seasons. As no other variables are selected as explanatory variables, it makes CLPP a sensitive soil biological indicator to in Regions with Mediterranean climate. A further analysis is required to strengthen the explanation, where attention should be given to more variables, more locations or conditions that are further controlled. Because we took into consideration the Autumn season as the soil moisture dry period and compared with the lower stress in Spring period, however, our work shows the concept of soil health to be of prime importance for a better understanding of soil behaviour.

## Supplementary Information

Supplementary Information.

## Data Availability

All data generated or analysed during this study are included in this published article [and its supplementary information files].
